# The Influence of Situational Cues on Children’s Creativity in an Alternative Uses Task and the Moderating Effect of Selective Attention

**DOI:** 10.3390/jintelligence8040037

**Published:** 2020-10-19

**Authors:** Marloes van Dijk, Elma Blom, Evelyn H. Kroesbergen, Paul P. M. Leseman

**Affiliations:** 1 Department of Pedagogy and Education: Development & Education of Youth in Diverse Societies, Utrecht University, Heidelberglaan 1, 3584CS Utrecht, The Netherlands; W.B.T.Blom@uu.nl (E.B.); P.P.M.Leseman@uu.nl (P.P.M.L.); 2 Department of Language and Culture, UiT The Arctic University of Norway, P.O. Box 6050 Langnes, NO-9037 Tromsø, Norway; 3 Behavioural Science Institute, Radboud University, P.O. Box 9104, 6500 HE Nijmegen, The Netherlands; e.kroesbergen@pwo.ru.nl

**Keywords:** creativity, stimulus environment, immediate situation, selective attention, perception–action perspective, affordances

## Abstract

Taking a perception-action perspective, we investigated how the presence of different real objects in children’s immediate situation affected their creativity and whether this effect was moderated by their selective attention. Seventy children between ages 9 and 12 years old participated. Verbal responses on a visual Alternative Uses Task with a low stimulus and high stimulus condition were coded on fluency, flexibility, and originality. Selective attention was measured with a visual search task. Results showed that fluency was not affected by stimulus condition and was unrelated to selective attention. Flexibility was positively associated with selective attention. Originality, net of fluency and flexibility, showed a main effect of stimulus condition in an unexpected direction, as children gave more original responses in the low stimulus condition compared to the high stimulus condition. A significant moderation effect revealed that children with better selective attention skills benefitted from a low stimulus environment, whereas children with weaker selective attention performed better in a high stimulus environment. The findings demonstrate differential effects of the immediate situation and selective attention, and support the hypothesis that creativity is impacted by immediate situation and selective attention, yet in unexpected ways.

## 1. Introduction

Research into children’s creativity has had a strong focus on individual differences and how these relate to cognitive functions. Only a few studies to date have addressed how children’s creative behaviour is influenced by characteristics of the situation in which this behaviour unfolds. Often, proposed definitions of creativity imply a conception of creativity as a stable psychological trait (Guilford 1950; Runco and Jaeger 2012), and typically offer no or only a limited account of how in particular rich stimulus environments can contribute to shaping creative behaviour. Building on research indicating that creativity varies across situations (Kletke et al. 2001; Warner and Myers 2010), the present study takes as a starting point the idea that creativity originates in individuals’ acts of perceiving, thinking, and acting upon stimuli and that these acts vary across individuals, situations, and time (see Orth et al. 2017 for a similar conception of creativity in creative motor action research). We hypothesise that children’s creativity depends on the array of stimuli present in the immediate situation and their ability to attend selectively to stimulus features allowing them to discover uncommon uses of everyday objects.

### 1.1. A Situated Approach to Creativity

Creativity is usually defined as the act of generating new ideas or establishing new connections between existing ideas or concepts (Sternberg 2006), and generally involves two aspects. The first aspect is related to the uncommonness of an idea (e.g., novelty, originality in various definitions) and the second one to the usefulness of the idea. Commonly used indicators of creative potential are divergent thinking tests which can be scored for fluency, flexibility, and originality (Reiter-Palmon et al. 2019). Fluency refers to the mere number of ideas generated. Measuring fluency, some researchers either include all responses or apply criteria of response adequacy to exclude repetition or incomplete answers (for a discussion, see Reiter-Palmon et al. 2019). Flexibility refers to the number of conceptual categories to which responses can be assigned, while originality refers to the uncommonness of specific responses. Originality can be determined either in an intersubjective way according to pre-established criteria (e.g., when a jury evaluates artistic products) or, most commonly applied, in an objective way where originality is based on the relative frequency of a particular idea in the whole sample (e.g., Forthmann et al. 2019). The three indicators are interrelated. Fluency can be regarded as facilitating the other two facets of creativity. Higher fluency increases the likelihood of generating an idea that can be assigned to another conceptual category (Nijstad et al. 2010) or is original (Forthmann et al. 2020; Silvia 2008). Flexibility can be similarly regarded as increasing the chance of generating original ideas by introducing category-switches (Nijstad et al. 2010). There is consensus that originality is at the core of the concept of creativity (Runco and Acar 2012; Runco and Jaeger 2012).

Most previous creativity research has been conducted within a classical trait theoretical framework, in which creativity is regarded as a psychological trait determining the degree of creativity of an individual’s behaviour across situations (Guilford 1950; Runco and Jaeger 2012). In line with this, explanations of individual differences in creativity are typically sought in cognitive differences, such as the ability to focus attention on relevant stimuli (Vartanian et al. 2007) or to inhibit prepotent but inappropriate (not original) responses (Stolte et al. 2019, or by differences in fluid intelligence, presumed to facilitate semantic memory search for remote, uncommon and thus original associations with a particular test stimulus (Beaty et al. 2014; Benedek et al. 2014a). In the trait framework, creativity is assumed to be relatively stable across situations (Guilford 1950; Runco and Jaeger 2012), while consequently the role of the immediate situation is a hitherto understudied aspect. However, evidence indicates that cross-situational stability is the exception rather than the rule. Studies have revealed only modest intra-individual stability of creativity test scores (Baer 1994; Jeon et al. 2011; Kupers et al. 2019), suggesting influence of situational characteristics. Evidence for the (at least partial) situatedness of creativity is reported in a number of experimental studies (see Davies et al. 2013, for a review on creative learning environments, Shalley et al. 2004, for a review on workplace creativity, and studies of Harrington 1975 and Lissitz and Willhoft 1985 focusing on instructional effects). These results indicate that creativity of an individual can be different across situations, an observation that is not often captured by classical trait views on creativity. In the next section, we will discuss the role of the immediate situation in greater detail. 

In accordance with recent views on perception, action, and cognition (Barab and Plucker 2002; Barsalou 2008; Glăveanu 2013; Osiurak et al. 2010; Wagman et al. 2016; Ye et al. 2009), the present study proposes that creativity is situated. Creative processes are influenced by objects, spaces, events, and their properties in the immediate situation through a person’s perceptions of and actions towards these situational factors (Barsalou 2008). Situations are generally information-rich and highly structured, and through this structured information an abundance of possibilities is offered to be acted upon (Boesch 2007). This abundance of action possibilities requires individuals to select the information most relevant for their current goal and to choose from all possibilities those actions that serve their goal optimally. Selecting actions involves attention mechanisms that mediate between bottom-up situational cues (signalling potential relevance) and top-down cognitive processes (biasing perception to select the information that fits a person’s goals; Cisek and Kalaska 2010). Attention is involved in many other cognitive processes, and is thought to be a core component of intelligence (Heitz et al. 2005; Kenett et al. 2016) and to underlie efficient memory retrieval (Silvia et al. 2013). Therefore, the current study included selective attention as a potential source of individual differences in children’s creative behaviour.

The framework we propose builds on Gibson’s (1979) ecological approach to human perception and cognition, in which the concept of *affordance* has been introduced to describe the relationship between individuals and their environments. An affordance is defined as the match between what a person currently is able to and interested in, on the one hand, and the relevant properties of the physical environment that trigger and enable particular actions of the person, on the other hand (Stoffregen 2003). For example, a fence affords jumping over it, but only for a person who has the appropriate leg length and an interest in reaching the other side of the fence. Affordances are specified by nested structures of subordinated affordances, which in turn are specified by perceivable properties such as surfaces, edges, rims, textures, and substances (Wagman et al. 2016; Ye et al. 2009). For example, the affordance drink-from-ability of a coffee cup is specified by the nested structure of the affordances grasp-ability, pour-in-ability, and contain-ability, which in turn are specified by combinations of perceivable invariant physical properties and qualities, also referred to as technical properties (Osiurak et al. 2010), such as the solidity and impermeability of the material of which cups are made, the roundness (of most cups), openness at the top, and closedness at the bottom. In normal everyday situations, individuals perceive the most common action affordances of objects based on typical, highly practised, and skilled use of these objects (Ye et al. 2009). For example, when looking at a coffee mug, the drink-from-ability will likely be perceived first. To detect alternative affordances for use, the nested subordinate affordances and object properties making up this common affordance have to be perceptually (or imaginatively) isolated from the fixed structure of the common affordance, foregrounded and made salient, and possibly recombined into a new structure specifying an uncommon affordance for use. For example, to detect that a coffee mug can be used as a template to draw a circle, one needs to perceive that the cup can be traced around. The presentation modality may affect the perception of common and uncommon affordances (Chrysikou et al. 2016; George and Wiley 2020). For example, Chrysikou et al. (2016) have shown that participants were more likely to perceive common affordances when presented visual vs. verbal stimuli in an open-ended Alternative Uses Task. In addition, the modality of presented examples influences creativity. Instructions that encourage individuals to avoid a set of common example ideas only enhance originality when the list is presented verbally, but not when examples are depicted visually (George and Wiley 2020). While the perception–action framework outlined here pertains to perceptual processing guided by outward-directed attention, we assume that similar processes can take place based on imagination or mental simulation, guided by an inward-directed focus of attention (Beaty et al. 2016; McClelland 2020; Van Dijk et al. 2018). 

To summarise, we presuppose that creative behaviour emerges from detecting alternative action affordances in the common affordance structures of everyday objects. Creativity is thus dependent on the stimuli in the situation (what is there to start with) and on the extent to which the individual explores, perceives, and exploits the (uncommon) action possibilities that the situation affords. Novel ideas and uses of objects can be discovered when individuals are able to attend to objects and object properties beyond the fixed nested affordances structures that specify common uses of objects and instead are able to single-out, foreground, and recombine nested affordances in new, uncommon structures. 

### 1.2. Creativity and the Immediate Situation

Defining creativity as the emerging skill of an individual to discover and combine affordances to act upon objects in novel ways, we predict that children’s creativity varies across situations due to (experimentally manipulated) situational differences. Previous research provides some support for this idea, although relevant research is sparse. In addition, research methods and outcome measures vary greatly across studies, and little research has investigated how the immediate situation impacts on the creative outcomes of children.

Previous research has shown that characteristics of the environment, such as the aesthetical quality of a workplace is positively related to creativity (Shalley et al. 2004). Additionally, studies conducted in 1970s and 1980s focusing on instruction effects in divergent thinking (e.g., Harrington 1975; Lissitz and Willhoft 1985) have shown situational influences, such as higher creativity after a simple instruction by the experimenter to be creative. Experimental studies demonstrated that school-aged children (Friedman et al. 1978) and students (McCoy and Evans 2002) in a setting with high creative potential were more original in making a creative collage than individuals in a setting with low creative potential. A setting with high creative potential, for example, is a room with multiple cues, such as objects that were built into the room intended to add aesthetic interest. A setting with low creative potential would be an almost empty room with only tables and chairs. The size of the physical space is also deemed important. The study of Chan and Nokes-Malach (2016) showed that participants in a larger room produced more novel alternative uses for everyday objects than participants who were assigned to a smaller room. According to the authors, the open environment of the large room stimulated participants to explore alternative uses more broadly. Guegan et al. (2017) investigated the extent to which contextual cues in a virtual environment affect creativity using Torrance’s Cardboard Boxes task (Torrance 1966). The creative performance of undergraduate students in a virtual creativity-conducive environment, a real meeting room, and its virtual replication were compared. Students in the creativity-conducive environment were found to be more original and elaborate in their answers than the other students. No differences were found in fluency and flexibility. Findings, however, are not consistent across studies, as Kletke et al. (2001) conclude in their review of studies on factors that support creativity and creative problem solving that the presence of stimuli in the immediate situation is related to higher fluency on creativity tasks.

Hardly any research has investigated how the immediate situation impacts on children’s creative behaviour. Besides the study of Friedman et al. (1978), the only study that examined the impact of the immediate situation has been conducted by Ward (1969) who compared responses of nursery school children on Wallach and Kogan’s (1965) Uses Task and Patterns Task, and the Instances Test in different environments. The study suggests that environmental factors do not affect all children in the same way, as children who were more creative produced more responses on the Instances Task in a cue-rich environment than in a cue-poor environment, while the fluency of children who were less creative did not vary across environments. The more creative children in this study used visual exploration of the objects present in the immediate situation as a key strategy (also see Runco and Chand 1995). First of all, it is important to investigate whether the finding that children’s creativity is impacted by the immediate situation can be replicated. Second, the study by Ward (1969) suggests there are individual differences with respect to the role of the immediate situation, but it is unknown which individual child characteristics contribute to children’s creativity, and how these characteristics interact with the presence of stimuli in the immediate situation. The current study addressed both issues. A possibly relevant child-internal factor that ties in with the situated view to creativity and that we further explored in our study, is selective attention. Selective attention is regarded as underlying more differentiated executive functions, such as shifting and inhibition. Especially in children, executive functions often appear as unitary, attention-based, instead of differentiated (Garon et al. 2008).

### 1.3. Creativity and Selective Attention

Selective attention refers to the type of processing involved in orienting attention towards specific stimuli while ignoring others (Stevens and Bavelier 2012). The competing information can occur externally (e.g., visual stimulation in the environment) or internally (e.g., thoughts or habitual responses). Selective attention draws on multiple attentional networks. Posner and Fan (2008) distinguish three attentional networks: orienting, alerting, and executive attention. Alerting attention refers to the ability to be prepared for effortful processing, and involves a constant vigilance to detect relevant information. Orienting attention refers to the capacity to direct attention to a potentially relevant stimulus. Executive attention is effortful and involved in the execution of goal-directed behaviours, and includes planning, selecting, and shifting among competing demands, maintaining purposeful behaviour, and monitoring the outcome. These three attentional networks are independent, but interrelated (Callejas et al. 2005; Mullane et al. 2016). Alerting attention can interrupt other attentional networks. It can activate the orienting network, drawing the attentional focus to a potentially relevant stimulus (Callejas et al. 2005), or modulate executive attention by prioritising processing of immediately relevant information (Weinbach and Henik 2012). Conversely, executive attention can bias alerting attention to be more sensitive to a specific input matching the current goal (Weinbach and Henik 2012). In addition, executive attention can deliberately steer orienting attention, directing the focus of attention to satisfy a specific goal (Mullane et al. 2016). 

In the present study, we focused on selective attention as possibly underlying individual differences in creativity. In the literature, selective attention is both used as a synonym for orienting attention (Schneider and Shiffrin 1977) and executive attention (Diamond 2013). Whether a selective attention task draws more on alerting, orienting, or executive attention is dependent on the specific task demands (Vandierendonck 2014). In the current study a visual search task was used, which is a perceptual task requiring attention to actively scan a visual display for a particular stimulus (the target) among other stimuli (the distractors). In this task, alerting attention is expected to be involved in being prepared to start processing the stimuli. While searching for the targets, attention shifts to potentially relevant stimuli (orienting attention), which is then evaluated to determine whether it matches the overarching task goal (executive attention). Within our situated perception–action approach to creativity, we propose that visual selective attention abilities enable children to focus on single affordances and their properties within the common affordance structure of an object, and to recombine singled-out affordances or the technical properties of affordances in novel ways, resulting in a creative response. It requires the basic capacity to deliberately direct attention in a top-down manner. Conceivably, children with better selective attention skills may detect these novel affordances more easily and faster than children with weak selective attention, and might therefore be more fluent, flexible, and original. Empirical studies on selective attention and creativity, all conducted within a classical trait theoretical framework, have concluded that better selective attention facilitates divergent thinking and creativity (Benedek and Fink 2019; Edl et al. 2014; Fink and Benedek 2014; Zabelina and Robinson 2010; Zabelina et al. 2016), whereas diffuse, broad-scope attention is related to real-world creative achievement (Ansburg and Hill 2003; Zabelina et al. 2016). Moreover, findings from the neuroscience literature suggest that task demands and attention jointly influence creativity (Beaty et al. 2014; Benedek et al. 2014b; Fink and Benedek 2014). For instance, Benedek et al. (2014b) have shown that shortening the exposure time to visual pictorial stimuli negatively affected creativity, but only when participants performed a creativity task dependent on sensory intake. It is however unknown how selective attention interacts with the amount of stimulation present in an immediate real-life situation when children are performing a creativity task.

### 1.4. The Present Study

The present study examined whether and how the immediate situation and selective attention are related to children’s creativity. Although the interaction effects of situational cues and individual characteristics on creativity have been studied before (Forthmann et al. 2019; Nusbaum and Silvia 2011; Nusbaum et al. 2014; Wilken et al. 2019), our study is one of the first to incorporate physical space and selective attention. To investigate creativity, children’s verbal responses in an Alternative Uses Task were analysed. In this task, children had to produce as many unusual uses of a target object in a limited amount of time. Adopting a perception–action approach, we hypothesised that creativity is dependent on the array of stimuli present in the immediate situation. Therefore, we expected that children in a high stimulus environment would outperform children in a low stimulus environment, and that their answers would be more fluent, flexible, and original. We also hypothesised that creativity is affected by the extent to which the individual explores, perceives, and exploits the (uncommon) action possibilities that the situation affords, actions for which attentional processes are relevant. We expected that children with high selective attention skills would be more creative (that is, more fluent, flexible, and original) than children with poor selective attention skills. This is consistent with research by Zabelina et al. (2016) suggesting that better selective attention is an advantage for divergent thinking and other types of creativity that involve test-based creativity. Finally, we explored a possible interaction between the richness of the immediate situation and selective attention, and tentatively predicted that children with better selective attention skills would benefit more from the presence of additional stimuli in the immediate situation than children with weaker selective attention skills when performing a creativity task. We did not have any predictions regarding specific or differential effects for fluency, flexibility, or originality.

## 2. Materials and Methods

### 2.1. Participants

Prior to data collection, a power analysis was conducted using G*Power 3 (Faul et al. 2007). We were interested in clear differences between the low-stimulus and high-stimulus conditions, to find support for our situated approach. With a power of 0.80 and an effect size of 0.40 (considered to be large, Cohen 1988), the minimum total sample to perform analyses of variance should be 52. A total of 70 children (41 girls) participated in the current study. Children were within classes randomly assigned to either the low (*n* = 36) or high stimulus (*n* = 34) condition. The mean age of the children was 11.07 years (*SD* = 0.69). They were recruited from five 5th and 6th grade classes of a regular primary school in a large city in the middle of The Netherlands. Regarding children’s background, socioeconomic status ranged between average and high, and about 20% of the children had a non-Dutch background, coming from immigrant or expat-families, which is representative of the modal primary school in The Netherlands (Statistics Netherlands 2020).

### 2.2. Materials

#### 2.2.1. Creativity

**Figure 1:**
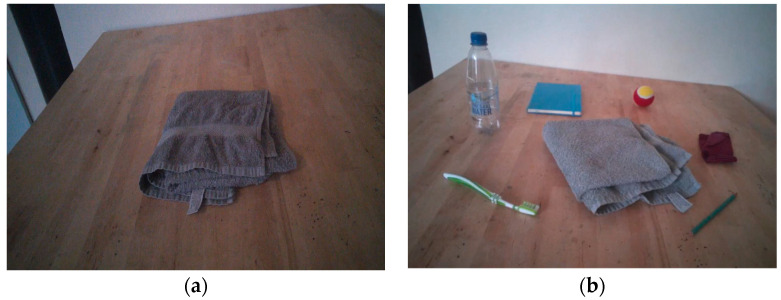
Participants’ view in the: (**a**) low stimulus condition; (**b**) high stimulus condition.

For the purpose of the current study, we adjusted the visual Alternative Uses Task of Agnoli et al. (2015) from a computer task to a real life task. This newly developed Alternative Uses Task required children to produce as many alternative uses of a target object within two minutes. The task consists of five trials that target the following objects: cap, pen, cloth hanger, spatula, and towel. We created a low stimulus condition and a high stimulus condition. In the low stimulus version of the task, only the target object was available for the children, see Figure 1. In the high stimulus version of the task, six other objects (tooth brush, water bottle, notebook, tennis ball, sock, and pencil) surrounded the target object, as demonstrated in Figure 1. All objects were randomly selected from an Alternative Uses Task database (Tanis et al. 2017). Children were told that the objects were present at the table, but were not specifically instructed to use the objects while performing the task. Eye movements were recorded by eye tracking glasses; the eye tracking data were collected for a second study aimed at a detailed investigation into children’s creative processes.

The present study focused on children’s verbal responses, which were coded for fluency, flexibility, and originality after exclusion of inadequate responses. To qualify as an adequate response, we applied the following criteria: verbal responses needed to be complete, understandable, and non-repetitive (i.e., similar responses should be counted only once). Furthermore, responses including the typical function of an object (e.g., *Using a pencil for writing*) were also excluded, since children were asked to produce *alternative uses* for an object. Of all responses, a proportion of 84% was considered adequate according to these criteria. Fluency was defined as the number of alternative uses that children produced. Flexibility was defined as the number of different conceptual categories that children used (e.g., *Using the pencil as a fork* and *Using the pencil as a knife* were considered to be instances of the same category, while *Using the pencil as a book mark* was coded as a different category). The semantic categories of the Torrance Tests of Creative Thinking (TTCT; Torrance 2008) were adapted to fit our study, see Appendix A for an overview of all conceptual categories. Originality was defined as the extent to which children produced infrequent alternative uses compared to the other children in the sample. An answer that was produced by less than 10% of the children in the sample was coded as original (e.g., *Making a bow and arrow out of the cloth hanger* was considered unoriginal, whereas *Using it as a shoehorn* was considered original, because this use was mentioned less than 10% of the times in the whole sample), see Appendix A for an overview of all unoriginal answers. A child’s originality score consisted of the sum score of all original answers that the child produced in the task. Ten percent of all data was independently coded on all creativity measures by a second rater; interrater reliability was considered satisfactory (*κ* = 0.75).

#### 2.2.2. Selective Attention

The subtest Sky Search of the Test of Everyday Attention for Children (TEA-Ch; Manly et al. 1999) was administered to assess children’s selective attention. According to Manly et al. (2001), the subtest has high reliability (test-retest correlation is 0.90) and convergent validity (e.g., correlation with Stroop Task is *r* = 0.44). The task consisted of an A3 sheet with 128 pairs of spaceships, 20 of which were identical. Children were instructed to search through the pairs of spaceships, and identify as quickly as possible all pairs in which both spaceships were identical by encircling the identical pairs. The task was timed with a stop watch. To control for drawing speed and children’s fine-motor skills, children had to encircle as fast as possible pairs of spaceships on a second A3 sheet with only identical pairs. The attention score of the Sky Search was calculated by subtracting the mean time per target (one identical pair of spaceships) spent encircling on the second sheet from the mean time per target spent encircling on the first sheet. Lower scores in this task indicated better performance. To facilitate the interpretation of the attention scores, we used the additive inverse, so that a higher score indicated better selective attention. 

### 2.3. Procedure

This research was approved by the ethical review board of the Faculty of Social and Behavioural Sciences of Utrecht University, the Netherlands. Active informed consent for all children was obtained from parents/caretakers prior to data collection. Children were told that participation was on voluntary basis, and that they could withdraw from the study at any point in time without having to explain the reason for withdrawal. All children were individually tested at their school in a quiet room where all possible distractors (e.g., posters, objects) were removed. The window was covered by a white sheet. They completed the test battery in one session of approximately 30 min. The Alternative Uses Task was the first task that was administered. Subsequently, a semi-structured interview about children’s task approach (not reported in this study), the Sky Search, and the Dutch version of the revised Early Adolescent Temperament Questionnaire EATQ-R (also not reported on in this study; Hartman and Rothbart 2000) were administered.

### 2.4. Data Analysis

Preliminary data analysis showed that two children had missing values on the creativity measures; their recording was unexpectedly interrupted. Their data were excluded from the analyses. Using *z*-scores, two univariate outliers were detected (one outlier on the high end on fluency, one on the low end on selective attention). Using Mahalanobis distance, no multivariate outliers were detected. To determine the effect of the outliers, analyses were conducted with and without outliers. As the results were impacted by the outliers, we decided to focus on the analyses with the outliers removed.

To assess the main effects of condition (low or high stimulus environment), selective attention, and the interaction effect between condition and selective attention on fluency, flexibility, and originality, a multivariate analysis of variance (MANOVA) was conducted (Section 3.1). Subsequently, univariate analyses of variance (ANOVAs) were conducted for the individual creativity measures. All assumptions were met: fluency, flexibility, originality, and selective attention scores were all normally distributed within the groups (non-significant Shapiro–Wilk tests and skewness and kurtosis), and the covariance matrices between the groups could be assumed to be equal (Box’s M test is non-significant, *p* = 0.67). The dependent variables were moderately to strongly intercorrelated, but did not exceed the *r* = 0.80 threshold of multicollinearity.

The first set of analyses (Section 3.2) did not reveal which of the three outcome measures (i.e., fluency, flexibility, and originality) was responsible for the overall effect of the interaction effect between condition and selective attention in the MANOVA, presumably because of the moderate-to-strong intercorrelations (see Table 1 and Table 2, for information on descriptive statistics and intercorrelations). As a second step (Section 3.3), we therefore decorrelated the dependent variables to be able to determine how flexibility (net of fluency) and originality (net of fluency and flexibility) were separately affected by the experimental condition and selective attention. To decorrelate the measures, we computed residual scores using regression analysis, partialling out the variance shared with the other measures. For flexibility, we partialled out the variance due to fluency (see Nijstad et al. 2010, who studied the confounding effect of fluency on flexibility). We composed the decorrelated flexibility score by letting fluency predict flexibility and saving the residual scores as the new (decorrelated) flexibility measure. The correlation between the decorrelated flexibility score and the original flexibility score was significant (*r* = 0.78, *p* < 0.01). For originality, we partialled out the variance due to fluency and flexibility (see Dumas and Dunbar 2014; Forthmann et al. 2020; Nijstad et al. 2010, for information on the confounding effect of fluency and flexibility on originality). Generating many ideas and switching between categories is not sufficient to generate original responses. In our study, we are focusing on the unique variance in originality beyond fluency and flexibility. We composed a decorrelated originality score by letting fluency and flexibility predict originality and saving the residual scores as the new (decorrelated) originality measure. The correlation between the decorrelated originality score and the original score was significant (*r* = 0.61, *p* < 0.01).

Furthermore, based on the results detailed below, we analysed the regions of significance (Bauer and Curran 2005) to further examine the statistically significant interaction effect between stimulus condition and selective attention on originality. 

## 3. Results

Descriptive statistics of the attention and creativity measures in the two conditions and in the overall sample are displayed in Table 1. The children in the low stimulus environment did not differ from the children in the high stimulus environment on sex, *χ*^2^(1, *N* = 70) = 0.86, *p* = 0.35, age, *t*(68) = −0.06, *p* = 0.96, or selective attention, *t*(68) = 0.51, *p* = 0.61. Intercorrelations between all variables can be found in Table 2. The three creativity measures were moderately to highly correlated. Note that Section 3.1 and Section 3.2 report the results of the analyses based on the untransformed scores, while Section 3.3 reports the findings based on the decorrelated scores.

### 3.1. Multivariate Analysis

A MANOVA was conducted to investigate the effects of condition and selective attention on creativity; the results are reported in Table 3. The multivariate effect of condition was not significant, *F*(3, 60) = 1.81, *p* = 0.15. The multivariate effect of selective attention was significant, *F*(3, 60) = 2.97, *p* = 0.04, $\eta^{2}$ = 0.13, indicating that there was an overall positive effect of selective attention abilities on the creativity measures. The overall MANOVA also returned a significant interaction effect between condition and selective attention on the combined creativity measures, *F*(3, 60) = 2.97, *p* = 0.04, $\eta^{2}$ = 0.13. This effect indicated that, depending on their selective attention skills, children responded differently to the amount of environmental stimulation (low versus high).

### 3.2. Univariate Analyses

Separate univariate analyses were conducted to explore the pattern of effects. The ANOVA for fluency and the ANOVA for originality did not return any significant main or interaction effect. The ANOVA for flexibility showed a significant effect of selective attention, indicating that children who scored better on selective attention were more flexible (i.e., used a higher number of different categories). No main effect of condition or significant interaction effect was revealed.

### 3.3. Univariate Analyses with the Decorrelated Measures

#### 3.3.1. Flexibility

We reran the ANOVA with the decorrelated measure of flexibility as the dependent variable and condition and selective attention as independent variables. This analysis yielded similar results as the previous ANOVA: a significant main effect for selective attention, *F*(1, 62) = 8.55, *p* < 0.01, $\eta^{2}$ = 0.12, no main effect for condition, *F*(1, 62) = 0.08, *p* = 0.77, and no interaction effect, *F*(1, 62) = 1.45, *p* = 0.22.

#### 3.3.2. Originality

**Figure 2:**
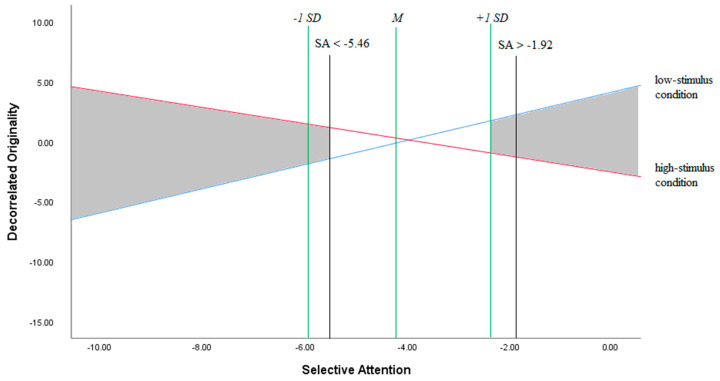
The interaction effect of stimulus condition and selective attention (SA) on decorrelated originality. The grey areas indicate statistical significance at *p* < 0.05 of the interaction effect.

We reran the ANOVA with the decorrelated measure of originality as the dependent variable and condition and selective attention as dependent variables. The main effect of condition was statistically significant with a medium effect size, *F*(1, 62) = 5.41, *p* = 0.02, $\eta^{2}$ = 0.08, indicating that children in the low stimulus environment outperformed children in the high stimulus environment. The main effect of selective attention was not significant, *F*(1, 62) = 0.29, *p* = 0.60. The interaction effect of condition and selective attention on originality was significant with a medium to strong effect size, *F*(1, 62) = 7.26, *p* = 0.01, $\eta^{2}$ = 0.11. The interaction effect is plotted in Figure 2, together with the regions of significance. Note that using the additive inverse of the raw scores of the selective attention measure, as explained in the Method section, resulted in negative values.

Figure 2 shows that the lower bound of significance (SA ≤ −5.46) is located between the mean score of selective attention (SA = −4.22) and one standard deviation below the mean (SA = −5.96). This indicates that the positive effect of the high vs. low stimulus condition on children’s originality is already significant at the moderately below-average level of selective attention. The higher bound of significance of the interaction effect (SA ≥ −1.92) is located beyond one standard deviation above the mean (SA −2.49), indicating that the positive effect of the low vs. high stimulus condition is only significant at a relatively high level of selective attention.

## 4. Discussion

Taking a perception–action perspective, the present study aimed to increase our understanding of how the immediate situation affects children’s creativity. More specifically, we investigated whether the presence of different objects in addition to the target stimulus would lead to more creativity compared to a situation with only the target stimulus. For this purpose, we designed an Alternative Uses Task with real objects and analysed children’s verbal responses in two conditions (low stimulus/without additional objects, high stimulus/with additional objects). In addition, we examined the effect of children’s selective attention on creativity and, in particular, if selective attention would moderate the effect of condition on creativity.

Our main findings can be summarised as follows. First, fluency was not affected by stimulus condition nor by children’s ability to attend selectively. Second, flexibility was also not affected by the stimulus condition, but the effect of selective attention turned out to be significant: children with better selective attention skills were more flexible in their answers. Third, originality was affected by both the stimulus condition and the interaction of stimulus condition and selective attention. In contrast to our hypothesis, however, children were more original in their responses in the low stimulus than in the high stimulus condition. This relationship was moderated by selective attention: children with better selective attention skills benefitted from the absence of additional objects and were relatively original in the low stimulus condition, whereas children with weaker selective attention performed relatively well in the high stimulus condition. Importantly, these effects on originality were only found for the decorrelated measure of originality, where the variance related to fluency and flexibility was partialled out. 

### 4.1. The Immediate Situation in Relation to Creativity

We presupposed that children’s creativity would be supported by the extent to which they explore, perceive, and exploit the multiple affordances that a situation affords, and expected that a high compared to a low stimulus situation would positively affect their fluency, flexibility, and originality scores. We did not find support for our hypothesis. Children in the high stimulus condition, on average, generated a similar number of verbal responses and showed a similar frequency of switching between semantic categories as children in the low stimulus condition. A possible explanation could be that these mere quantitative measures conceal that the thinking processes, and resulting responses and category switches, may have been qualitatively different between the stimulus conditions (and perhaps more original in the high stimulus condition). Indeed, closer inspection of the verbal responses may suggest that children tended to use qualitatively different thinking processes in the two conditions. While many responses in the high stimulus condition seem to originate from perceptual associations between matching action affordances (such as “*Using a spatula to play hockey*” in the trial with the spatula as target object, while the child concerned was looking at the tennis ball as one of the other presented stimuli), indicating an outward focus of attention and perception-based processing, the responses in the low stimulus environment seem to reflect an inward focus of attention drawing upon semantic and episodic memory (“*Using a towel to catch flies*” in the trial with the towel as target object, possibly because this particular child remembered such an action from a past experience). Thus, the immediate situation may have influenced individual responses qualitatively, but did not result in quantitative differences. Future research should take these possible qualitative differences in underlying processes into account. As a starting point, the recently developed coding scheme of Matheson and Kenett (2020) could be useful, which defines six dimensions (i.e., analogy vs. action, whole vs. parts, same vs. different, concrete vs. abstract, novel vs. familiar, and towards vs. away) to characterise the strategies used by participants to generate divergent thinking responses.

Yet, despite these indications of qualitatively different thinking processes as a consequence of the stimulus condition, also the results for originality (with the variance of fluency and flexibility partialled out) did not support the expectation that children in a high stimulus environment would outperform children in a low stimulus environment. On the contrary, children in the low stimulus condition turned out to be more original than children in the high stimulus condition. A possible, and perhaps likely, explanation is that in an Alternative Uses Task the presence of real, visible stimuli is neither a sufficient nor necessary condition for discovering uncommon uses of everyday objects, thus contradicting our perception–action account of creativity. Indirect support for this explanation comes from a study by Benedek et al. (2014b). In this study, an Alternative Uses Task was presented in a condition in which all stimuli were kept visible during the entire task and a condition in which the stimuli were masked after 500 ms. No difference in originality was found between the two conditions. Thus, to be creative in an Alternative Uses Task, the (prolonged) presence of visible stimuli may not be as important as we expected. This, however, does not explain the advantage that children in general seem to experience in the low stimulus condition.

An additional, or possibly alternative, explanation, which can also explain the higher originality in the low stimulus condition, relates to our sample-based “relative” coding of originality. Originality scores were based on the frequency of particular uses that were mentioned by the children relative to all uses that the children in the current sample mentioned. If a particular use was mentioned less than 10% of the times in the sample of all mentioned uses, it was coded as original. Children in the high stimulus environment were all presented with the same array of six stimuli at all trials with only the target stimulus changing over trials and, consequentially, mentioned relatively often similar uses. For example, using the spatula as a bookmark was coded as unoriginal, because several children in the high stimulus condition came up with this fairly uncommon use of the spatula (which presumably was discovered in relation to the booklet that was part of the stimulus array). Put differently, although uncommon uses discovered in the high stimulus condition may have been original in an absolute sense, they were not original in a relative sense due to the fact that the array of stimuli was the same for all children in this condition and influenced their thinking processes in a similar way. Conversely, children in the low stimulus condition more often based their answers on personal experiences and idiosyncratic memories, which were mostly not shared by other children and, therefore, more likely to be scored as original, although not necessarily original in an absolute sense (e.g., using the target object towel to extinguish a fire). This is in accordance with previous research (Forthmann et al. 2017; Harrington 1975) suggesting that task demands may influence creativity. For instance, Forthmann et al. (2017) instructed participants explicitly “to be creative”, however, this instruction unintendedly biased the outcomes. As a consequence of the instruction, responses mentioning common uses were inhibited, with the remaining ones being relatively rare, thus original, while responses mentioning uncommon uses occurred more often, relatively, and were therefore less original. Therefore, we recommend considering alternative ways of coding in future research which are not based on the relative response frequency within the sample, such as using a jury to assess the responses.

Finally, although the children in the high stimulus condition were not specifically instructed to use the surrounding objects on the table while performing the task, it is possible that they have felt obligated to use the objects in their answers. As a result, they might have systematically tried to include these objects in their responses in outward perception-based thinking processes instead of using also inward memory-based processes and imagination in combination with perceptual processing. This may have had a limiting effect on their originality. Evidence for such a constraining effect can also be found in previous work of George and Wiley (2020) and Smith et al. (1993) on the use of examples of alternative uses as part of the task instruction. In both studies, participants, upon performing the actual task, were found to search for alternative uses similar to the examples, resulting in less original responses.

There may have been other constraining effects of the stimulus array. In the high stimulus condition of the present study, the degree to which the objects were semantically related varied between pairs. Semantically strongly related pairs (such as *towel–tooth brush*) may have induced functional fixedness, impeding thinking about novel uses of these objects (Duncker 1945). Functional fixedness seems to occur more when visual instead of verbal stimuli are used (Chrysikou et al. 2016). Note, however, that in the current study, there were no systematic differences in fluency, flexibility and originality between the trials, although trials varied in potential functional fixedness of stimulus pairs. In other research, working with a theoretical model of creativity based on spreading activation in semantic memory networks, similar effects were found when priming subjects too strongly on a particular stimulus. For example, in the study of Friedman et al. (2003), students completed visual tasks that forced them to focus their perceptual attention on either a broad or narrow visual area. Narrow initial focusing resulted in less original responses in a task where subjects were asked to think of a creative title for a photograph, presumably due to limited spreading of activation through the memory network. 

### 4.2. Selective Attention in Relation to Creativity

Selective attention was found to have differential effects on fluency, flexibility, and originality. To understand these differential effects, we need to consider both the nature of selective attention as measured in this study and the type of thinking processes involved in performance on the Alternative Uses Task. The visual search task used in this study as a measure of selective attention is primarily tapping into effortful executive attention: the effortful top-down process of finding relevant prespecified stimuli while ignoring perceptually similar distractors. The absence of a relationship between selective attention and fluency replicates the findings of Zabelina and Robinson (2010), and may indicate that the mere quantity of responses is not or not strongly dependent on thinking processes that require effortful executive attention. It is likely that fluency scores mainly represent relatively effortless, automatically generated responses involving semantically close memory- or stimulus-based associations (Bai et al. in review; Gilhooly et al. 2007). Shifting between semantic categories, our measure of flexibility, in contrast, requires deliberate and effortful deactivation and reallocation of attentional focus, which may explain the main effect of selective attention on flexibility: children with better selective attention generated answers that switched more flexibly between semantic categories. 

Contrary to our expectations, there was no main effect of selective attention on originality (net of fluency and flexibility). However, there was an interaction effect of selective attention with stimulus condition. We hypothesised that children with better selective attention would benefit more than children with weaker selective attention from a high stimulus environment with regard to originality, because a high vs. low stimulus environment would provide them with more opportunities to perceive uncommon affordances, for example by changing the salience of particular nested affordances or by perceptually singling-out particular object features and recombining them with other features to discover uncommon uses of objects. However, our results revealed the opposite. There are several possible explanations. Children with better attention skills may have relied more on inward-oriented thinking processes and used selective attention for deeper, effortful search in their memory networks (cf. Beaty et al. 2014). The presence of additional stimuli may not have been supportive and could even have been distracting to this type of processing. Children with weaker selective attention, on the other hand, may have been less capable in using effortful inward-oriented memory-based processes for finding uncommon uses and, therefore, were more dependent on the additional cues provided in the high stimulus condition. In this case, weaker top-down selective attention may even have been an advantage allowing for stronger bottom-up processing within a broader attentional scope. 

The interaction effect of stimulus condition and selective attention was significant when the decorrelated measure of originality was used, that is, when the variance in originality related to fluency and flexibility was partialled out. A possible explanation, already touched upon above, is that particularly fluency (accounting for the largest part of the shared variance with originality) may be based on a different type of thinking process than the process that leads to more original responses. Suggestive evidence comes from studies on the serial order effect of originality in divergent thinking and creativity, referring to the phenomenon that the first responses to a particular stimulus in divergent thinking or creativity tasks like the one used in the current study come fast but are overall less original, while the later responses in a series come at a slower pace but are more original (Beaty and Silvia 2012; Gilhooly et al. 2007; Wang et al. 2017). Studies on the serial order effect, using think-aloud verbal reports by subjects explaining how they came up with a particular use, found that the vast majority of uses mentioned by children were generated based on relatively effortless memory associations (Gilhooly et al. 2007). As associative processing likely exploits the network of close associations of a stimulus first, this possibly explains why these uses were overall less original (cf. Bernard et al. 2019). A much smaller number of the mentioned uses, mainly occurring toward the end of a series, indicated according to the children’s reports of the involvement of effortful processing and these responses were on average rated as more original. Thus, the fluency measure may mainly reflect responses based on associative processing, while concealing the much smaller count of responses based on effortful executive processing that are likely to be more original. When the variance of fluency and flexibility are partialled out, as done in the current study using a decorrelated measure of originality, the originality score corresponds strongly with effortful creative processes. Only this “purified” measure of originality shows effects of stimulus condition and selective attention. In addition, individual differences in how a creativity task is approached may play a role. For many children, higher fluency goes hand in hand with higher originality simply because a larger quantity of responses increases the probability of producing original responses. However, originality is not fully dependent on fluency. A fluent child could produce many responses that lack originality. Some children produce only a few responses which, however, are mostly original. Independence may also apply to the relationship between originality and flexibility. Again, the likelihood of an original response may be higher if a child makes a category switch, but category switches are not required for original response. For example, whereas one participant in our sample made eight category switches and produced two original responses (out of eleven), another participant only made three category switches and produced twelve original responses (out of thirteen). By partialling out the variance in originality that is related to mere fluency and flexibility, individual differences in originality surface.

There are several limitations to the current study. First, the originality measure of the current study was a measure based on statistical frequency within the sample, which may have favoured originality scores in the low stimulus condition as we argued. Furthermore, frequency estimates for scoring responses as original are more accurate with larger sample sizes (Reiter-Palmon et al. 2019). Very low-frequency estimates have been recently shown to suffer from a lack of measurement precision when the estimates are based on a small sample (Forthmann et al. 2019). In the current study, we observed a clear bimodality in the frequencies of original and unoriginal responses. The original answers were mostly mentioned by only 5% or less of the children, while most of the unoriginal responses were produced by 15 to 20% or more, which is clearly above the 10% threshold. However, to avoid a lack of measurement precision, intersubjective rating of originality or automated scoring is recommended in future research on creativity (for more information, see Beaty and Johnson 2020 or Dumas et al. 2020). Second, the task instruction may have primed the children in the high stimulus condition too strongly to outward perceptual processing while a mix of outward and inward memory-based processing could have promoted originality better. Unfortunately, we do not know how the children came up with their responses. Especially for the high stimulus condition, it would have been interesting to know to what extent the children made use of the additional stimuli in their responses. Prompting children to explain how they came up with an idea would be valuable to obtain insight into their thinking process (e.g., Bai et al. in review). Finally, we only used selective attention as a measure of the executive processes involved in creativity. Although this measure did provide new insights into how selective attention is related to creativity, other executive functions are also deemed important for explaining individual differences in creative behaviour (Benedek et al. 2014a; Pan and Yu 2016; Stolte et al. 2019; Zabelina et al. 2019). Executive functions, such as shifting among competing demands or inhibiting irrelevant stimuli, may also influence creativity in interaction with varying stimulus environments. We recommend including several measures of executive functions in future research examining the joint effect of situational and individual characteristics on creativity.

To conclude, the present study highlighted the variability of creativity across situations. Although in the opposite direction of what was expected, the presence of stimuli in the immediate situation was found to influence creativity: children were more original in the low stimulus condition compared to the high stimulus condition. We found support for a moderating effect of selective attention on the relation between stimulus environment and originality: Children with better selective attention skills benefitted from a low stimulus environment, whereas children with weaker selective attention performed better in a high stimulus environment. Although the current findings, as such, do not support the perception–action view on creativity we proposed at the outset of this study, several findings indicate that perceiving environmental cues impacts on creativity. These indications include the type of thinking processes occurring during the Alternative Uses Task and the interaction effect of the stimulus environment with selective attention. Further research on the combined effects of situational characteristics and individual capabilities on creativity is recommended. In this research, several issues need to be considered: the nature of the thinking processes involved, the way in which originality scores are determined, and the kind of measures that are used to assess individual capabilities.

## Figures and Tables

**Table 1:** Descriptive statistics for Fluency, Flexibility, Originality, and Selective Attention.

	Low Stimulus Condition (*n* = 34)		High Stimulus Condition (*n* = 34)		Total (*n* = 68)	
	*M*	*SD*	*M*	*SD*	*M*	*SD*
Fluency	27.52	11.52	22.59	11.62	25.01	11.75
Flexibility	9.47	2.26	7.91	2.26	8.69	2.38
Originality	15.03	8.63	11.65	6.53	13.34	7.78
Selective Attention	−4.26	1.75	−4.19	1.74	−4.22	1.73

**Table 2:** Intercorrelations between Fluency, Flexibility, Originality, and Selective Attention.

	1.	2.	3.	4.	5.
1. Fluency	_				
2. Flexibility	0.63 **	_			
3. Originality	0.72 **	0.74 **	_		
4. Selective Attention	−0.05	0.31 *	−0.22	_	
5. Decorrelated Flexibility	0.00 ^a^	0.78 **	0.33 **	−0.33 **	_
6. Decorrelated Originality	0.00 ^a^	0.00 ^a^	0.61 **	−0.06	0.00 ^a^

* *p* < 0.05, ** *p* < 0.01; ^a^ as the variance from fluency and/or flexibility was removed in the decorrelated measures, the correlation with the untransformed fluency and flexibility was zero.

**Table 3:** Multivariate Analysis of Variance of Factors Condition, Selective Attention, and Interaction Effect of Condition and Selective Attention on Fluency, Flexibility, and Originality.

Source	Wilks’ Lambda	*F* (*df*)	*p*	$\eta^{2}\;$
Condition	0.92	1.81 (3, 60)	0.15	0.08
Fluency		0.13 (1, 62)	0.72	0.01
Flexibility		<0.01 (1, 62)	0.99	<0.01
Originality		2.47 (1, 62)	0.12	0.04
Selective Attention	0.87	2.97 (3, 60)	0.04	0.13
Fluency		0.24 (1, 60)	0.62	<0.01
Flexibility		6.89 (1, 60)	0.01	0.10
Originality		2.53 (1, 60)	0.12	0.04
Condition × Selective Attention	0.87	2.97 (3, 60)	0.04	0.13
Fluency		0.07 (1, 60)	0.79	<0.01
Flexibility		1.27 (1, 60)	0.27	0.02
Originality		1.10 (1, 60)	0.30	0.02

## Appendix A

**Table A1:** Overview of all conceptual categories.

Conceptual Category	Example
Art use	To paste things on
Carrier	To put stuff in
Construction use	Brick
Container	To hold something
Costume	Clothing
Cover	To cover something
Destruction	To throw away
Ecological use	To recycle
Games	To throw at
Household appliances	Eating utensils
Music/noise makers	Musical instrument
Protection	Shield
Scientific uses	To experiment
Support	Book ends
Tools	Scoop
Toys	Telephone
Weapon	To attack

**Table A2:** Overview of all unoriginal answers.

Cap	Pen	Cloth Hanger	Spatula	Towel
Arts and crafts	Cleaning	Bow and arrow	Balancing	Arts and crafts
Catching things	Clip	Cleaning	Bending	Blanket
Cleaning	Cutlery	Clamp	Bookmark	Carrier
Container	Destroying	Destroying	Catapult	Carpet/rug
Decorating	Disassemble	Fishing rod	Cleaning	Cleaning
Destroying	Hurting someone	Painting	Crushing	Clothing
Drawing on it	Making music	Throwing	Destroying	Covering
Eating utensil	Using the ink	Throwing out	Hitting someone	Curtain
Frisbee		Use the hook	Making music	Decoration
Painting		Weapon	Playing tennis	Destroying
Playing a game		Zipline	Scoop	Flag
Storage			Scratcher	Hiding
Throwing out				Hitting
				Mat
				Painting
				Pillow case
				Stuffed animal
				Table cloth
